# Piezoelectric Ceramics with High d_33_ Constants and Their Application to Film Speakers

**DOI:** 10.3390/ma14195795

**Published:** 2021-10-03

**Authors:** Sowon Kim, Heechul Lee

**Affiliations:** Department of Advanced Materials Engineering, Korea Polytechnic University, Siheung 15073, Korea; swkim919321@gmail.com

**Keywords:** piezoelectricity, soft relaxor, low-temperature sintering, multilayer piezoelectric actuator, film speaker

## Abstract

A multilayer piezoelectric material was fabricated using piezoelectric materials with low-temperature sintering capabilities and high piezoelectric coefficients to develop a functionally superior piezoelectric speaker with a large-displacement deformation. A soft relaxor was utilized to prepare the component materials, with the optimized composition of the investigated piezoelectric ceramics represented by $0.2Pb\left({\left(Zn_{0.8}Ni_{0.2}\right)}_{\frac{1}{3}}Nb_{\frac{2}{3}}\right)O_{3}-0.8Pb\left(Zr_{0.5}Ti_{0.5}\right)O_{3}$. $Li_{2}CO_{3}$ was added to assist the low-temperature sintering conducted at 875 °C, which yielded a multilayer piezoelectric material with superior properties ($d_{33}\;$= 500 pC N^−1^, $k_{p}\;$= 0.63, $g_{33}\;$= 44 mV N^−1^). A multilayer piezoelectric actuator with a single-layer thickness of ~40 µm and dimensions of 12 × 16 mm^2^ was fabricated by tape casting the prepared green sheets. Finite element analysis revealed that the use of a PEEK film and a smaller silicone–rubber film as a composite in the diaphragm realized optimal frequency-response characteristics; the vibrations generated by the piezoelectric element were amplified. The optimal structure obtained via simulations was applied to fabricate an actual piezoelectric speaker with dimensions of 20 × 24 × 1 mm^3^. The actual measurements exhibited a sound pressure level of ~75 dB and a total harmonic distortion ≤15% in the audible frequency range (250–20,000 Hz) at an applied voltage of 5$\;V_{p}$.

## 1. Introduction

The demand for ceramic components with superior reliability and functionality has increased recently to keep pace with the enhanced performance of various electronic devices including smartphones, personal computers, and wearable devices. Small, thin, and highly efficient internal electronic components are being developed to integrate numerous functions in a high-density manner within the limited dimensions of mobile technological products [1,2].

In this regard, several complex electronic components have been replaced with piezoelectric materials. The development of acoustic components, which are essential parts of electronic products such as communication and information broadcasting equipment, is particularly gaining in attention [3,4]. Currently, commercialized dynamic speakers use magnetic diaphragms and coils, which limits their miniaturization; moreover, these current-driven systems consume significant amounts of power. Piezoelectric speakers, which are actuators that convert electrical energy into acoustic energy, have emerged as an alternative to conventional speaker technologies. Piezoelectric speakers generate sound by using the vibration of diaphragms caused by the mechanical deformation of piezoelectric materials upon the alternating voltage; therefore, these speakers are thin, lightweight, and consume low amounts of power [5,6].

The application of piezoelectric materials to acoustic actuators necessitates the development of materials with high piezoelectric constants and conversion efficiencies [2,7,8,9]. Because the property of the actuator that causes displacement is proportional to its piezoelectric constant, the higher the value, the easier it is to implement in an acoustic actuator. Three-component ceramics, which are solid solutions of Pb-based perovskite-type relaxors, have a high solid-solubility-limit and can be used to fabricate piezoelectric materials by varying their piezoelectric coefficients, depending on the application. Therefore, the development of materials for acoustic actuators must involve the preparation of base materials with improved properties such as relaxor ferroelectrics based on lead zirconate titanate ($Pb\left(Zr_{x}Ti_{1-x}\right)O_{3}$ (0 ≤ *x* ≤ 1); PZT) [10,11,12]. In addition, the applications of multilayer piezoelectric components are expanding to achieve lower driving voltages compared to those of single-layer piezoelectric materials [13,14]. Low-temperature sintering can assist in fabricating a multilayer piezoelectric actuator. Given that the ceramic layer and the inner electrode are fired simultaneously during the fabrication of multilayer piezoelectric actuators, low-temperature sintering can enable the use of electrodes with low melting points and relatively low costs, which increases the price competitiveness [15,16]. In addition, low-temperature sintering can prevent issues that frequently occur in high-temperature sintering such as the degradation of properties caused by changes in the compositions of materials due to the volatilization of PbO and structural deformations such as bending and cracking [17].

When the diaphragm is used as a metal or alloy plate in the existing piezoelectric speaker, sharp and rough sound is generated, and the manufacturing process of attaching the piezoelectric component to the diaphragm is difficult. Moreover, the piezoelectric polymer speaker has an extensive diaphragm area because its piezoelectric coefficient is not high. Hence, it is not suitable to replace the existing dynamic speaker [18,19]. In order to solve these problems, we have attempted to make a smaller piezoelectric speaker that can reduce sound distortion by using highly flexible and elastic diaphragm materials.

Therefore, the development of piezoelectric materials that can cause prior displacement deformation and be subjected to low-temperature sintering was investigated in this study by examining the effects of the changes in the amount of an added dopant and the ceramic composition ratio on their piezoelectric properties; soft relaxor materials such as $Pb\left(Ni_{1/3}Nb_{2/3}\right)O_{3}\;$(PNN) and $Pb\left(Zn_{1/3}Nb_{2/3}\right)O_{3}$(PZN) were employed as substitutes [20,21]. Device design and analysis were performed using finite element analysis software (ATILA FEA, Micromechatronics, USA) with the properties of the optimized piezoelectric material used as inputs. A multilayer piezoelectric actuator was subsequently fabricated to realize the optimized, simulated structure by layering a Ag electrode with a small amount of Pd and a PZNN-PZT piezoelectric material. Finally, components such as the diaphragm and frame were assembled to fabricate an acoustic actuator, whose acoustic properties were evaluated according to frequency.

## 2. Methods

### 2.1. Preparation of the Piezoelectric Materials

High-purity powders (>98%) sourced from Kojundo Chemical Laboratory were used in the experiments. $PbO,\;ZrO_{2},\;\;TiO_{2},\;ZnO,\;\;Nb_{2}O_{5}$, and NiO were used as starting materials to synthesize piezoelectric materials using a general solid-state reaction method. The powders were weighed according to various composition ratios of $xPb\left({\left(Zn_{0.8}Ni_{0.2}\right)}_{1/3}Nb_{2/3}\right)O_{3}-\left(1-x\right)Pb\left(Zr_{0.5}Ti_{0.5}\right)O_{3}$ (PZNN-PZT, x = 0.10–0.30). To ensure even dispersion, the powders were mixed with zirconia balls in an ethanol solution for 24 h and dried for more than 4 h at 120 °C. The resulting powders were subsequently subjected to two-stage calcination [22]. The $Li_{2}CO_{3}$ dopant was mixed with the fabricated powders for 24 h by varying its concentration from 0.1 wt.% to 0.4 wt.%. An appropriate amount of an aqueous solution of 10 wt.% polyvinyl alcohol (PVA) was added to the powder as a binder, and the powder was molded into a Ø 10 mm disk by applying a pressure of approximately 10 MPa. Cold isostatic pressing was carried out using a water pressure of 130 MPa to ensure that the molded ceramic had a denser structure. Sintering was subsequently conducted in a temperature range of 850–950 °C for 4 h. The crystallographic structures of the sintered specimens were analyzed by X-ray diffraction (XRD; BRUKER, Billerica, MA, USA), and the microstructures were examined by scanning electron microscopy (SEM; Nova Navo SEM 450, ThermoFisher, Waltham, MA, USA) to estimate the degree of densification. The average particle sizes were calculated by drawing several parallel lines on the SEM images using the linear intercept analysis method. The number of grains intersecting the parallel lines were counted from the images, and the average grain size was determined by dividing the length of the parallel lines by the number of grains intersecting the lines. The electrical properties were investigated by grinding both sides to a thickness of 1 mm, and applying and heat treating Ag electrodes at 650 °C for 30 min. For poling, a 2.5 kV mm^−1^ electric field was applied in a silicon oil bath at 120 °C for 30 min, and the electrode was aged in air for 24 h [23]. A $d_{33}$ meter (Model YE2730, HANTECH, Gunpo, Korea) and an impedance analyzer (Model 4990A, Keysight Technologies Inc., Santa Rosa, CA, USA) were used to calculate the piezoelectric coefficient ($d_{33}$), piezoelectric voltage constant ($g_{33}$), and electromechanical coupling factor ($k_{p}$) according to the IEEE standard method on polarized specimens. The polarization–electric-field (P–E) hysteresis loops were analyzed using a TF analyzer (TF-2000E, aixACCT, Aachen, Germany) to observe the changes in polarization with the application of an external electric field.

### 2.2. Preparation of Multilayer Ceramics

A multilayer piezoelectric actuator for piezoelectric speakers was fabricated using the optimized composition from the aforementioned experiments. A PZNN-PZT powder developed for the green-sheet production of a multilayer piezoelectric actuator was mixed with ethanol, a solvent containing methyl ethyl ketone, and a dispersant in a ball mill for 24 h. Subsequently, a binder (polyvinyl butyral) was added and ball milling was repeated to produce a slurry, which eventually yielded an ~40-µm-thick green sheet by tape casting. The electrode paste was prepared by mixing 80 wt.% Ag and 20 wt.% Pd paste with a small amount of the PZNN-PZT powder, a binder (ethyl cellulose), and a plasticizer in a three-roll mill. The green sheet produced in the preceding step was punctured with a hole for pattern printing, and an electrode pattern corresponding to each layer was formed by screen printing. A total of seven layers were stacked and subsequently laminated at room temperature under a pressure of 0.04 MPa for unification. After printing the electrodes for the top and bottom parts of the laminated sheet, they were cut by component units, and terminations were performed on the edge parts to connect the corresponding layers [24,25]. Sintering was performed in a belt-type furnace, with high-temperature co-fired ceramic sheets placed on the top and bottom of the sample to prevent bending; a constant load was applied to the top side during sintering. The sample was subsequently fired for approximately 15 min at a final sintering temperature of 875 °C to fabricate the multilayer piezoelectric actuator.

### 2.3. Fabrication of the Piezoelectric Speaker

A 12 × 16 × 0.32 mm^3^ multilayer piezoelectric actuator was used to fabricate piezoelectric speakers containing diaphragms made of polyetheretherketone (PEEK) and silicone rubber films with different physical properties and dimensions. A highly stiff stainless-steel frame that effectively absorbed the vibrations of the piezoelectric diaphragm and did not cause unnecessary vibrations was used as the frame, with epoxy used as the adhesive between each material for stable bonding. The acoustic properties of the piezoelectric speaker were investigated by attaching it to the center of a baffle plate in an anechoic chamber with a sound insulation performance of 30 dB(A) and an acoustic wedge to block external noise. The distance between the reference microphone and the speaker was fixed at 10 cm. The total harmonic distortion (THD) properties and the sound pressure level (SPL) output in the audible frequency range (250–20,000 Hz) were measured using an audio analyzer (FX-100, NTi, Schaan, LI, Liechtenstein) at an applied voltage of 5 $V_{p}$.

## 3. Results and Discussion

In order to develop a piezoelectric material capable of sintering at a low temperature and having a piezoelectric constant, the composition ratio of PZNN and PZT and the amount of $Li_{2}CO_{3}$ doping material were varied. 

**Figure 1:**
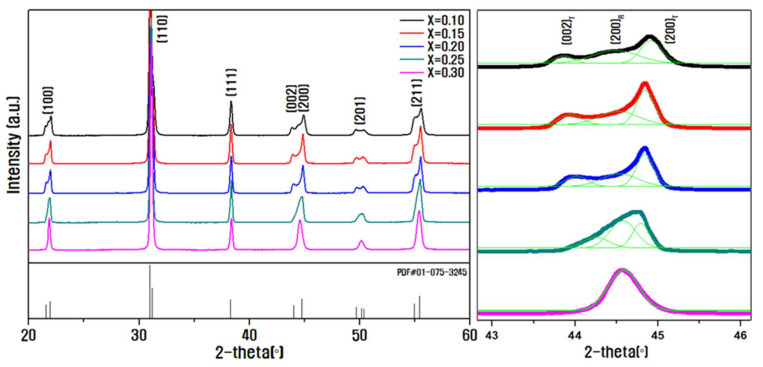
X-ray diffraction (XRD) patterns of $xPb\left({\left(Zn_{0.8}Ni_{0.2}\right)}_{1/3}Nb_{2/3}\right)O_{3}-\left(1-x\right)Pb\left(Zr_{0.5}Ti_{0.5}\right)O_{3}$ ceramics with varying contents of PZNN.

Figure 1 shows the XRD patterns of the ceramics sintered at 900 °C with varying composition ratios of PZNN and PZT without the addition of $Li_{2}CO_{3}$; the results indicate the formation of stable perovskite structures without the production of secondary phases in all of the samples. The enlarged diffraction patterns in the 2θ range of 43°–46° indicates the occurrence of a phase transition from tetragonal to rhombohedral phases with an increase in the molar fraction of PZNN; moreover, a large (200)R rhombohedral diffraction phase appeared between the (002)T and (200)T tetragonal peaks [26,27]. The compositions corresponding to *x* = 0.15–0.25 represent the morphotropic phase boundary (MPB) region, in which the rhombohedral and tetragonal phases are mixed to a certain extent. The PZNN component is known to prefer rhombohedral symmetry, and the increase in the PZNN molar fraction causes a change in the crystal lattice [10]. The rhombohedral phase becomes predominant at a composition of *x* = 0.30.

**Figure 2:**
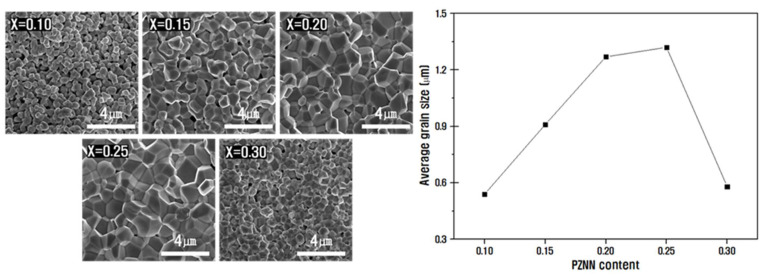
Field emission scanning electron microscopy (FE-SEM) images of the fractured surfaces of the $xPb({\left(Zn_{0.8}Ni_{0.2}\right)}_{1/3}Nb_{2/3})O_{3}-\left(1-x\right)Pb\left(Zr_{0.5}Ti_{0.5}\right)O_{3}$ ceramics with varying concentrations of PZNN, and the calculated corresponding average particle sizes.

Figure 2 shows the SEM images of the fracture-surface microstructures of the PZNN-PZT ceramics sintered at 900 °C with varying composition ratios. The images revealed that the microstructure densification proceeded with an increase in the PZNN content from 0.1 to 0.25; moreover, the average crystal grain size increased from 0.5 µm to 1.3 µm without the formation of pores [28]. The increase in grain size was expected to vary with the composition ratio, and the particle growth behavior was anticipated to affect the piezoelectric properties [29,30]. Zheng et al [31], suggested that the decrease in particle size promotes the enhancement of the internal stress and stabilizes the rhombohedral phase; however, the Figure 1 results indicate the occurrence of a complete phase transition to the rhombohedral phase due to the decrease in particle size at a composition of *x* = 0.30 [32,33].

**Figure 3:**
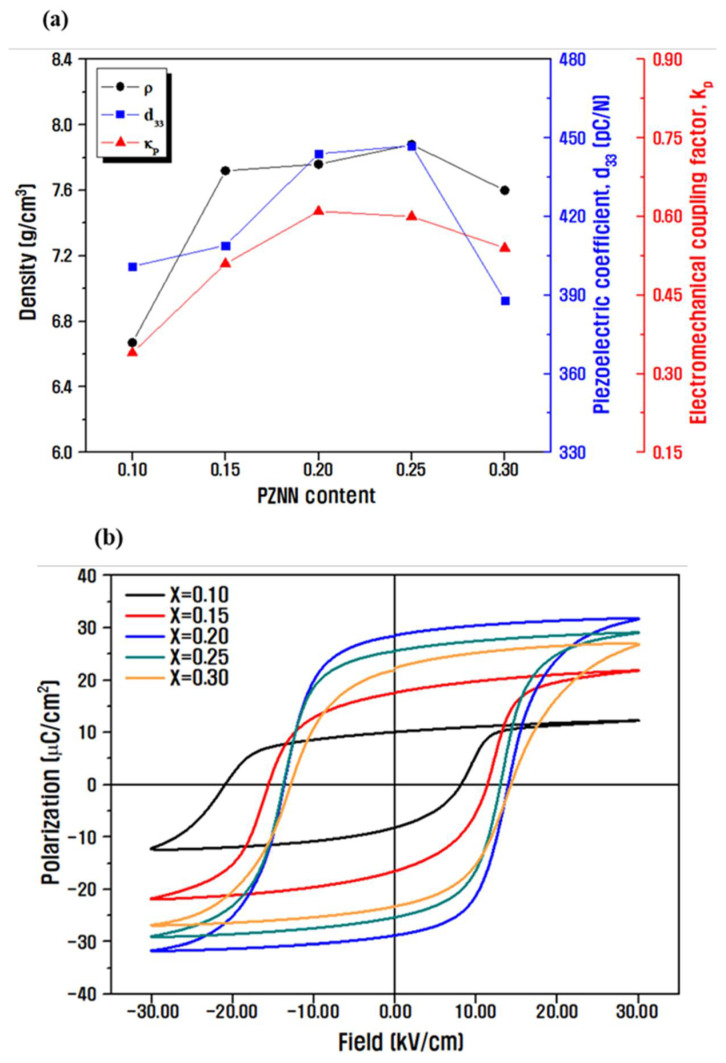
Measurements of the (**a**) densities and electrical properties ($d_{33}$, $k_{p}$) of the ceramics sintered at 900 °C with different contents of PZNN, and (**b**) the corresponding polarization–electric-field (P–E) hysteresis loops.

Figure 3 shows the measured densities and electrical properties of the prepared ceramics with different composition ratios. The density increased with an increasing content of PZNN up to *x* = 0.25 (Figure 3a) because of the increase in crystal grain size and the decrease in the number of pores, as shown in the SEM cross-sectional images in Figure 2 [34,35]. The highest density value of 7.92 g cm^−3^ corresponds to a relative density of approximately 97% when the theoretical density value of 8.1 g cm^−3^ in the PZNN-PZT ceramic was applied. $d_{33}$ and $k_{p}$ also changed in proportion to the grain size and density, and these piezoelectric properties presumably improve with an increase in density because of the lack of empty spaces in crystal grains that do not contribute to the piezoelectric properties. Figure 3b shows the measured P–E hysteresis loops of the ceramics with different compositions of PZNN and PZT at a frequency of 1 Hz. The highest remnant polarization corresponds to 2·$P_{r}$ = 56 µC cm^−2^ for x = 0.20, which can be related to the polarization efficiency based on the crystal structure. In general, dipoles can be rearranged during polarization by 6, 8, and 14 crystallographic orientations in the axial direction of the tetragonal phase, the rhombohedral phase, and the MPB region with the two coexisting phases, respectively. The highest remnant polarization at the aforementioned composition corresponds to the MPB region because the polarization efficiency is maximized and a strong piezoelectric effect is exhibited due to the facilitated rearrangement of the domain wall [36]. The grain boundary phases were confirmed to increase with decreasing grain size, and the value of the remnant polarization decreased under application of the same electric field due to the clamping effect of the domain wall [11,31,37,38].

**Figure 4:**
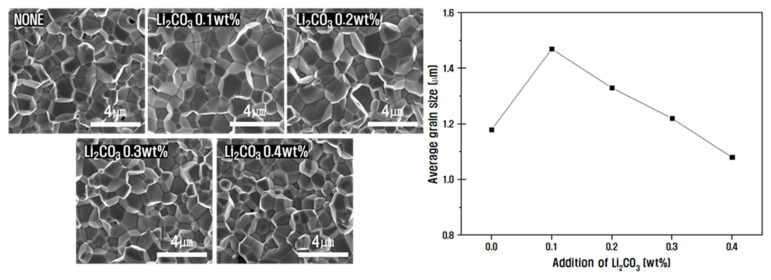
FE-SEM images of the fractured surface of PZNN-PZT ceramics with varying amounts of the $Li_{2}CO_{3}$ dopant and the corresponding estimated average particle sizes.

When the sintering temperature varied in the range of 850 to 950 °C, the lowest temperature for crystallization of the 0.2PZNN-0.8PZT was 875 °C. Figure 4 shows the images of the fractured surface of the PZNN-PZT ceramics with varying amounts of the $Li_{2}CO_{3}$ dopant at a sintering temperature of 875 °C. The particle size increased from 1.18 µm to 1.47 µm upon the addition of 0.1 wt.% $Li_{2}CO_{3}$, presumably because of the low melting point of $Li_{2}CO_{3}$ (723 °C), which induced the formation of a liquid phase during sintering and densified the ceramic particle surface [39,40,41]. However, the further addition of $Li_{2}CO_{3}$ (≥0.2 wt.%) decreased the particle size due to the formation of the $Li_{2}PbO_{3}$ secondary phase upon the addition of $Li_{2}CO_{3}$ above the solid solubility limit; moreover, the grain boundary segregation at the triple junction in the crystal interferes with particle growth [42,43]. Therefore, the addition of a small amount of $Li_{2}CO_{3}$ (0.1 wt.%) can enable its use as a sintering agent for low-temperature sintering by densifying the particles without forming a secondary phase.

**Figure 5:**
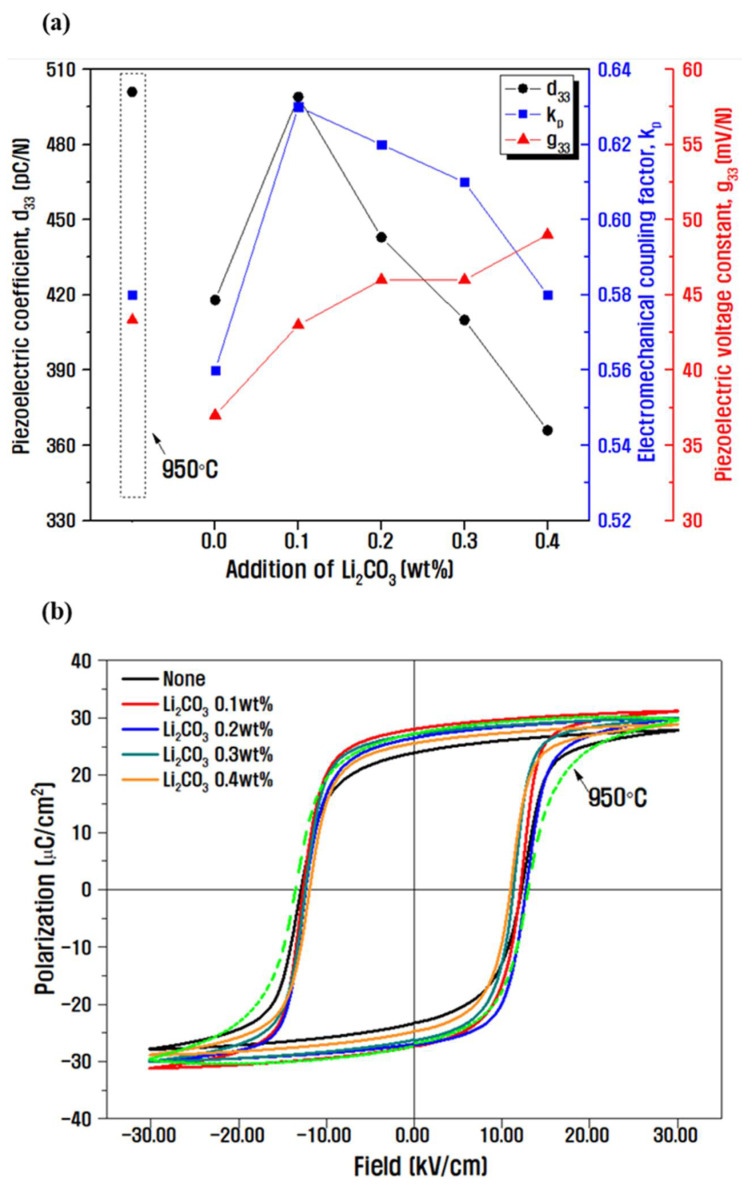
(**a**) Electric properties ($d_{33}$, $k_{p}$, $g_{33}$) of specimens sintered at 875 °C with varying amounts of the $Li_{2}CO_{3}$ dopant, and (**b**) the corresponding P–E hysteresis loops. The sample sintered at 950 °C without doping is represented by the data within dotted lines in (**a**) and the green, dashed profile in (**b**).

Figure 5 shows the electrical characterization results of the PZNN-PZT ceramics with different doping amounts of $Li_{2}CO_{3}$ sintered at 875 °C. Doping the low-temperature-sintered sample with 0.1 wt.% $Li_{2}CO_{3}$ was confirmed to yield superior or similar values of $d_{33}$ (500 pC N^−1^) and $k_{p}$ (0.63) compared to the characteristic values of 501 pC N^−1^ and 0.58 obtained at a higher sintering temperature of 950 °C (Figure 5a). Additionally, its density value was as high as 8.0 g cm^−3^, which was similar to that of the sample sintered at 950 °C. However, the values of these piezoelectric properties decreased upon the further addition of $Li_{2}CO_{3}$ (≥0.2 wt.%). This is due to the replacement of $Li^{+}$ (0.76 Å) with $Zr^{4+}$ (0.72 Å) or $Zn^{2+}$ (0.74 Å), which have similar ionic radii and higher valences, thereby forming an oxygen vacancy for charge compensation. These oxygen vacancies induce space charge, which suppresses the movement of the domain wall and plays a role in hardening the material. This increases the content of Li and reduces the values of $d_{33}$ and $k_{p}$ [16,40,43,44]. The piezoelectric voltage coefficient ($g_{33}$), which is an important physical property for enabling high displacement and low driving voltage in multilayer actuator applications, can be estimated using the equation $d_{33}$/($\varepsilon_{0}\cdot \varepsilon_{r}$), according to which a $Li_{2}CO_{3}$ doping of 0.1 wt.% corresponds to a $g_{33}$ of 43 mV N^−1^, a value similar to that obtained at a sintering temperature of 950 °C (44 mV N^−1^). $g_{33}$ exhibited a sustained increase with increasing concentration of $Li_{2}CO_{3}$, possibly because of the decrease in $d_{33}$ at *x* ≥ 0.2 wt.% and the significant decrease in permittivity [8,45]. The P–E hysteresis loops (Figure 5b) suggest that the addition of 0.1 wt.% $Li_{2}CO_{3}$ at a sintering temperature of 875 °C (2·$P_{r}$ = 56 µC cm^−2^, $E_{c}$ = 1.2 kV mm^−1^) leads to a larger remnant polarization and a lower coercive electric field, indicating the increasing likelihood of polarization with the addition of the dopant compared to that in the ceramics sintered at 950 °C without $Li_{2}CO_{3}\;$(2·$P_{r}$ = 52 µC cm^−2^, $E_{c}$ = 1.3 kV mm^−1^) [46]. Finally, an excellent piezoelectric material capable of sintering at a low temperature was developed by adding 0.1 wt.% $Li_{2}CO_{3}$ to the optimized composition of 0.2PZNN-0.8PZT.

The structure of the piezoelectric speaker with optimal characteristics was simulated prior to its fabrication by performing simulations using modal and harmonic functions in the ATILA piezoelectric analysis software. A low fundamental resonance frequency is crucial for obtaining improved acoustic properties of a speaker over the entire investigated frequency range. The fundamental frequency ($f_{0}$) is expressed by the following equation: $f_{0\;}$= $\sqrt{k/m}$ (k = elasticity coefficient, m = mass) [47,48], which suggests that the elasticity coefficient of the diaphragm of the speaker considerably influences the frequency response characteristics. Therefore, the acoustic characteristics of diaphragm materials with different physical properties were analyzed. Table 1 shows the dimensions and driving conditions of different materials that were used to construct speakers via the piezoelectric simulation analysis. Prior to the analysis, the structure of the piezoelectric speaker was modeled. The multilayer piezoelectric actuator was designed with dimensions of 12 × 16 × 0.32 mm^3^ and a single 40-µm-thick piezoelectric layer (seven layers in total). The values of $d_{33}$ and $k_{p}$ of the piezoelectric layer were added as inputs, which were obtained from the measured values of the previously optimized sample. In order to improve the acoustic performance over the entire investigated frequency range, 0.1-mm-thick silicone rubber and 0.05-mm-thick PEEK film with different vibration responses were used as diaphragm materials; moreover, 0.4-mm-thick stainless-steel frame was used, and these layers were placed at the top and bottom of the diaphragm. The analysis conditions were set to predict the frequency response characteristics and displacement range in a frequency range of 250–20,000 Hz at a drive voltage of 5 $V_{p}$.

**Figure 6:**
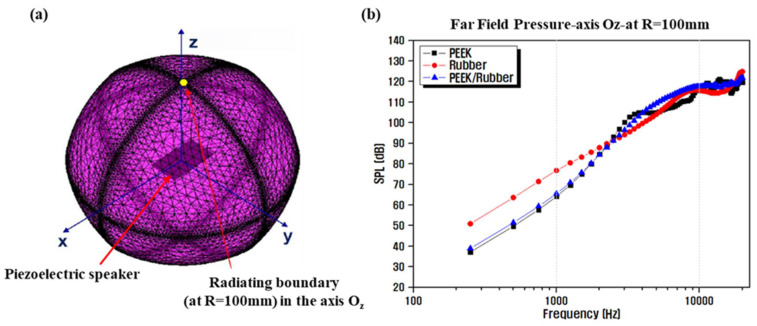
(**a**) Finite element model for the numerical analysis of the investigated piezoelectric speaker, and (**b**) the calculated frequency response characteristics of the acoustic diaphragms of the piezoelectric speaker.

Figure 6 shows the geometry of the model designed for the finite element analysis of the piezoelectric speaker, along with the results of the piezoelectric simulation with respect to the diaphragm materials. The displacement characteristics of the acoustic diaphragms, depending on their frequencies, are proportional to the output sound pressure of the piezoelectric speaker and can be regarded as characteristics related to the output sound pressure. The output SPL is the ratio of the sound pressure (P) to the reference sound pressure ($P_{0}$) expressed on a logarithmic scale $P_{0}$= $20\;e^{-6}$ Pa for airborne sound waves, which is based on the minimum auditory threshold at 1 kHz. As shown in Figure 6a, the values of pressure, P, at 100 mm in the z-axis direction of the acoustic center were obtained and substituted into the equation, 20·log10(P/$P_{0})$, to examine the degrees of the outputted SPLs ($P_{0}$ = $20\;e^{-6}$ Pa, reference pressure for air) [49]. Figure 6b shows that the use of the PEEK single film (E = 3.76 GPa) as the diaphragm material satisfies the high-frequency-band acoustic characteristics due to its low elasticity and high rigidity; however, the sound pressure in the low-frequency band is definitely low. In contrast, the use of a thick silicone-rubber film (E = 0.001 GPa) with a low Young’s modulus as a single diaphragm material leads to an increased vibration absorption rate due to its superior flexibility, which partially absorbs the distortion components generated by the vibrations and improves acoustic features in the low-frequency range [50,51]. Attaching the relatively thin PEEK film to the rubber film results in a slight reduction in the low-frequency-band sound pressure due to the use of a thicker diaphragm. Nevertheless, the output SPL characteristics improved up to a relatively high frequency range, which confirms that the insufficient vibration characteristics of two films can be complemented by laminating films with different vibration characteristics.

**Figure 7:**
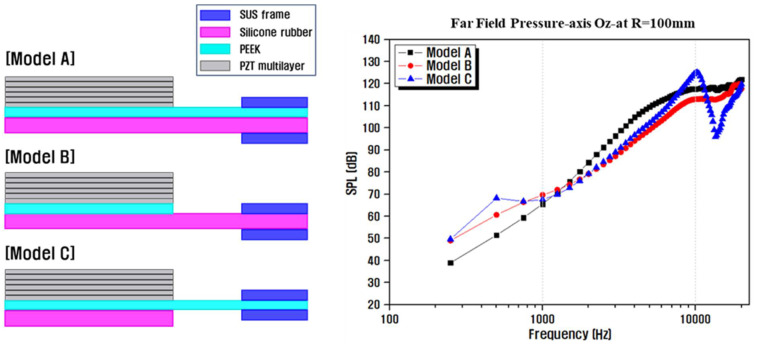
Simulated frequency responses of piezoelectric speakers constructed using acoustic diaphragm structures of different sizes.

Figure 7 shows the results of the simulation analyses performed using different dimensions or structural designs of the two connected diaphragm types; the PEEK/silicon-rubber composite film used in the aforementioned FEA simulations was denoted as Model A. The smaller PEEK layer was attached to the center of the diaphragm in Model B, which exhibited more flexible characteristics than those of Model A, resulting in improved low-frequency SPLs [50]. In contrast, the Model C structure with a smaller silicon rubber film attached to the center of the diaphragm was found to act as a soft mass at the core where vibrations were generated, resulting in relatively uniform improvements in the output SPL characteristics in the medium-frequency region [5,47]. The Model C structure exhibited optimal characteristics with an average output sound pressure of approximately 101 dB (at 10 cm) in the entire frequency range, although a large dip occurred at approximately 13 kHz.

**Figure 8:**
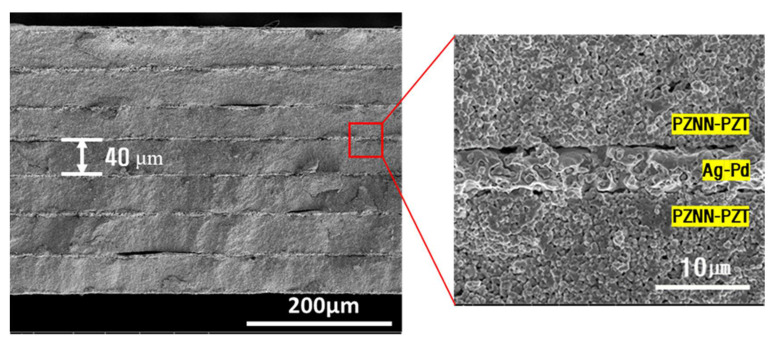
SEM images of the fractured surface of the co-fired multilayer PZNN-PZT/Ag–Pd actuator.

Figure 8 shows an SEM image of the cross-section of the multilayer piezoelectric material fabricated by tape casting of the 0.2PZNN-0.8PZT piezoelectric powder with 0.1 wt.% $Li_{2}CO_{3}$ dopant), which was previously optimized through experimental analysis of the fabrication of the piezoelectric speaker. A total of seven ~40-µm-thick layers were visible in the multilayer piezoelectric material, similar to that in the simulation design. The enlarged view of the ceramic and electrode layers showed almost no interdiffusion based on the well-defined classification of each layer [24]; moreover, the sintering of the ceramic layers was relatively adequate, considering the use of a low sintering temperature of 875 °C. In addition, the process control for the heat treatment leads to the absence of structural defects after sintering, despite the thinness of the piezoelectric material in an overall thickness of approximately 320 µm.

**Figure 9:**
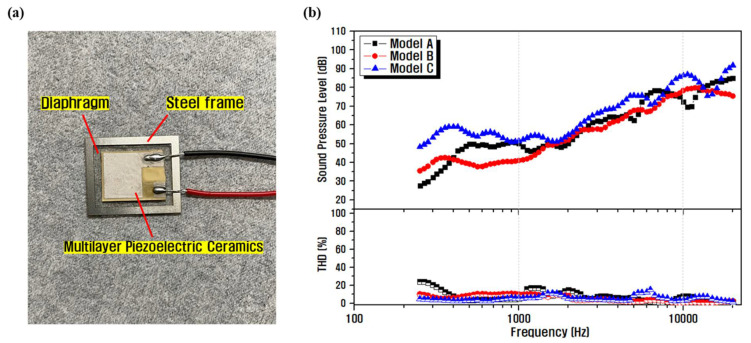
(**a**) A representative fabricated piezoelectric speaker. (**b**) SPL and THD characteristics of the fabricated speaker.

A representative piezoelectric speaker fabricated with a diaphragm based on the simulated results is shown in Figure 9, along with the estimated results of the SPL and THD properties with respect to frequency. The piezoelectric speaker shown in Figure 9a was fabricated by first attaching PEEK and silicone rubber with epoxy, fixing the soldered piezoelectric material to the center of the combined composite film diaphragm with epoxy and letting it harden, before finally covering it with a steel frame. The actual measurements (Figure 9b) show that the average output SPL of the Model C structure was estimated to be 75 dB (at 10 cm) at an applied drive voltage of 5 $V_{p}$; moreover, the THD was less than or equal to 15% over the entire measured frequency range, which was superior to that of speakers with different structures (Model A: 70 dB, Model B: 67 dB). Model C also showed a dip due to resonance at 14 kHz in the actual measurement, similar to the simulated results. The response characteristics also showed a similar trend across the investigated frequency range compared to the simulated results; however, the output SPL showed an overall decrease. This is presumably because of the process deviations that occur during speaker assemblies such as non-uniform epoxy application and inaccurate positioning of the acoustic diaphragm, or property degradation caused by differences in the piezoelectric properties of the single sintered ceramic sample and the multilayer piezoelectric sintered sample. 

In the present work, we developed a multilayer piezoelectric actuator using the optimized 0.1 wt.% $Li_{2}CO_{3}$ doped PZNN-PZT material capable of sintering at a low temperature. Additionally, a piezoelectric speaker with an acoustic diaphragm structure in which two different films were laminated is suggested, showing the feasibility as a next-generation speaker to replace the existing dynamic speaker. Therefore, further improvements in the acoustic properties can be achieved by reforming the process of fabrication, which can lead to the piezoelectric actuator being utilized for various applications.

## 4. Conclusions

In this study, we designed, prepared, and characterized a miniatured piezoelectric speaker with improved acoustic performances using a multilayer piezoelectric actuator and highly flexible and elastic diaphragms. The optimization of low-temperature sintered materials was conducted herein to develop materials for piezoelectric speakers capable of causing large-displacement deformation by adjusting the mixing ratio of PZNN and PZT, and varying the amount of $Li_{2}O_{3}$ dopant in $Pb\left({\left(Zn,Ni\right)}_{1/3}Nb_{2/3}\right)O_{3}-Pb\left(Zr,Ti\right)O_{3}$. A composition ratio of 0.2PZNN-0.8PZT and 0.1 wt.% $Li_{2}O_{3}$ dopant assisted in realizing the optimal properties of density (8.0 g cm^−3^), $d_{33}$ (500 pC N^−1^), $k_{p}$ (0.63), $g_{33}$ (43 mV N^−1^), and 2·$P_{r}$ (56 µC cm^−2^). A multilayer piezoelectric ceramic actuator was fabricated using this optimized ceramic powder by the tape casting method; moreover, the actuator was stably sintered at a low temperature of 875 °C and exhibited a thickness of only 320 µm. A piezoelectric speaker with dimensions of 20 × 24 × 1 mm^3^ was fabricated by attaching the multilayer piezoelectric ceramic to a PEEK/silicone-rubber composite film diaphragm, which was optimized using an FEA-based piezoelectric analysis software. An operating voltage of 5 $V_{p}$ in the 250–20,000 Hz frequency range led to stable SPLs with an average SPL of approximately 75 dB and THD less than or equal to 15% in the speaker with the composite diaphragm constructed using PEEK and a smaller silicone-rubber layer. Further improvements in the acoustic properties of the ultra-thin (≤1 mm) piezoelectric speaker fabricated herein can be achieved by developing additional fabrication processes for eventual use in small and lightweight electronic devices such as smartphones and tablets.

## Figures and Tables

**Table 1:** Constituent materials and analysis conditions for the ATILA simulation of the piezoelectric speakers.

Parameter		Conditions
Geometry (mm)	Multilayer piezoelectric actuator	12 × 16 × 0.32
	Silicone rubber	0.1
	PEEK	0.05
	Steel frame	20 × 24 × 0.4
Definition	Input voltage (V_p_)	5
	Frequency range (Hz)	250–20,000

## Data Availability

The data presented in this study are contained within the article.
